# Undescribed and imperiled vertebrate biodiversity near an American urban center

**DOI:** 10.1098/rsbl.2024.0652

**Published:** 2025-04-23

**Authors:** Chase D. Brownstein, Daemin Kim, Julia E. Wood, Zachariah D. Alley, Maya F. Stokes, Thomas J. Near

**Affiliations:** ^1^ Department of Ecology and Evolutionary Biology, Yale University, New Haven, CT, USA; ^2^ Department of Biology, Texas A&M University, College Station, TX, USA; ^3^ Edge Engineering and Science, Houston, TX, USA; ^4^ Department of Earth, Ocean, and Atmospheric Science, Florida State University, Tallahassee, FL, USA; ^5^ Department of Ecology and Evolutionary Biology, Yale University, New Haven, 06511 USA, USA; ^6^ Yale Peabody Museum, New Haven, CT 06511, United States of America

**Keywords:** biodiversity, endangered, conservation systematics, Birmingham, darter, hotspot

## Abstract

Urban expansion threatens biodiversity hotspots and endemic species. In this study, we describe two imperiled new species of fishes belonging to the vermilion darter (*Etheostoma chermocki*) complex. These new species are restricted to individual stream systems surrounding the city of Birmingham, Alabama, USA, and are at risk of extinction due to anthropogenic development. Genomic species delimitation reveals that members of this species complex, which differ subtly but consistently in meristic counts and coloration, show high levels of genomic divergence and little gene flow among them. These brilliantly coloured species, whose diversification tied to the erosional dynamics of the Black Warrior River basin, exemplify the imperiled, yet undescribed, species diversity within an urban landscape in the southeastern North American biodiversity hotspot.

## 1. Introduction

Anthropogenic pollution and habitat degradation are inducing global declines in species diversity (e.g. [1,2]), and a quarter of all freshwater species are now at risk of extinction [3]. The southeastern North American freshwater biodiversity hotspot includes the most species-rich freshwater biotas outside of the tropics [4–9]. This region is home to several species-rich endemic vertebrate clades, including darters (*Etheostomatinae*) [10–13], minnows (*Pogonichthyinae*) [14–20] and madtoms (*Noturus*) [21–23]. Much of the area that makes up this biodiversity hotspot remains unprotected [4,24], and freshwater species in this region show among the highest rates of extinction and decline of any freshwater biota across the globe [7,25–28].

The delimitation and description of species form the foundation for conservation efforts. However, many southeastern North American freshwater clades remain understudied. Although *Pogonichthyinae* and *Etheostomatinae* rank among the top 20 most species-rich subfamilies of fishes and are known to contain numerous undescribed species [10,29–35], these clades have experienced some of the lowest rates of species discovery in the past decade [36]. The limited protection of critical aquatic habitats, coupled with a slow pace of species description, hinders the conservation and protection of the exceptional freshwater biodiversity of southeastern North America.

**Figure 1.**
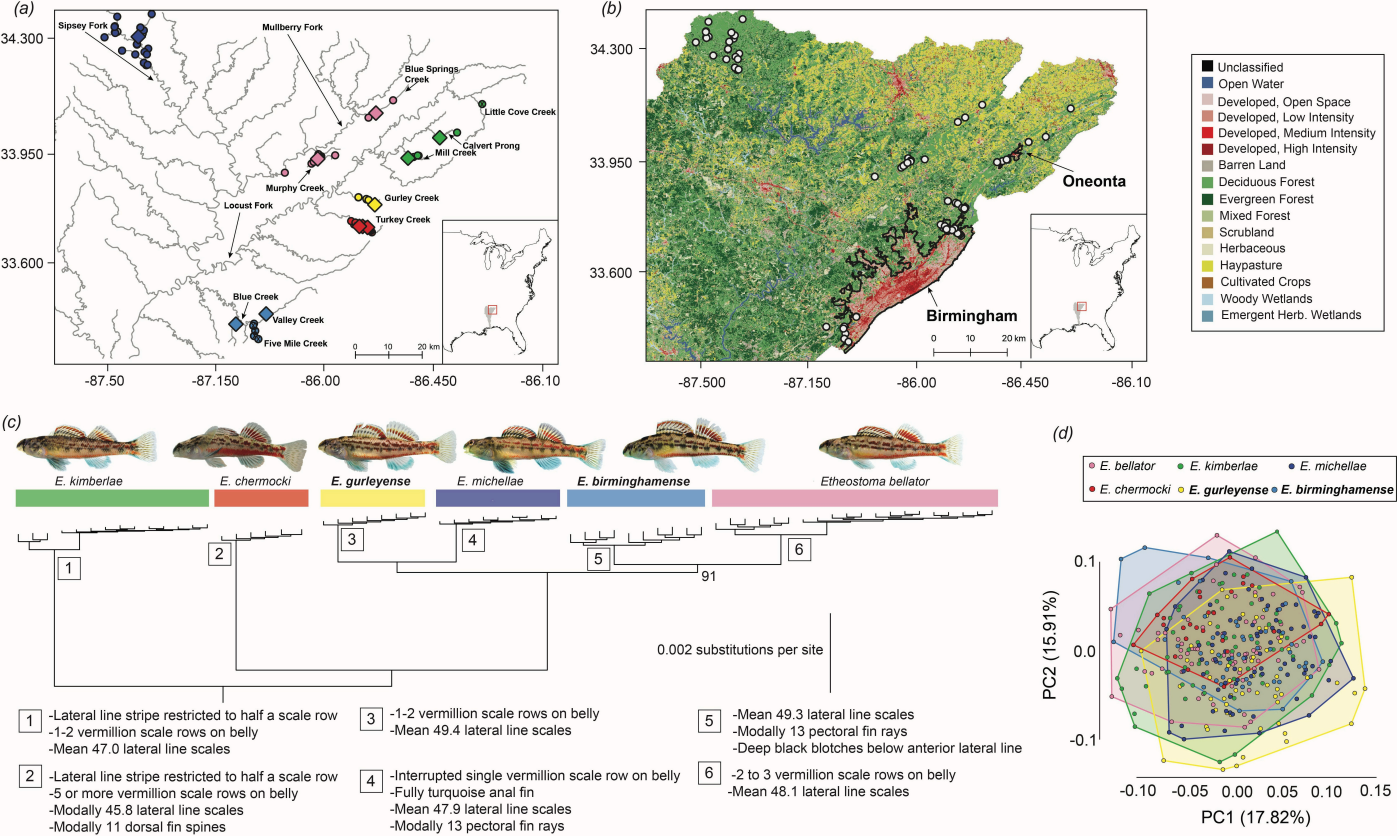
Four new imperiled darter species from Alabama. (*a*) Map illustrating the distributions of the species in the *Etheostoma chermocki* complex in the Black Warrior River system of Alabama, USA; dots represent collection localities curated from Fishnet2.net. Diamonds denote collection sites of specimens that were sequenced for ddRAD loci. X-marks on dots denote localities from which corresponding darter species are extirpated. The inset shows the location of the distribution map in the Mobile Basin. (*b*) Land cover data map for 2021 for the region shown in (*a*); land cover data from [37]. White dots represent collection localities curated from Fishnet2.net, and urban areas of Birmingham and Oneonta, Alabama, are outlined with a black border. (*c*) The phylogeny of the *Etheostoma chermocki* complex based on the ddRAD dataset presented in [30], with outgroups removed, highlighting diagnostic features of each new species. Bolded species names are new species. Photographs are by J.E.W., Z.D.A., and Bernie Kuhajda (used with permission). Numbers at nodes indicate bootstrap supports <100 (intraspecific nodes are not labelled). Numbers in boxes refer to the table of diagnostic morphological characters. (*d*) Plot of the first two principal components from the principal components analysis of 377 individuals for nine meristic traits.

**Figure 2.**
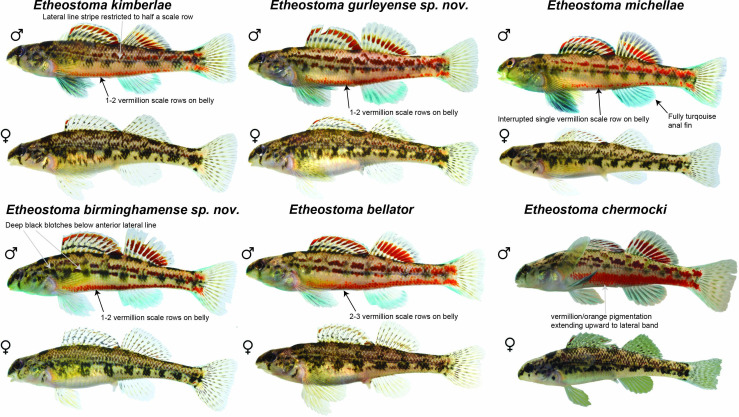
Comparative coloration of the new darter species. Comparison of coloration and pigmentation of *Etheostoma kimberlae, Etheostoma gurleyense* sp. nov. *Etheostoma michellae*, *Etheostoma birminghamense* sp. nov., *Etheostoma bellator*, and *Etheostoma chermocki*. Photographs are by J.E.W., Z.D.A., and Bernie Kuhajda (used with permission).

Here, we describe two new species of darters from the US state of Alabama (figures 1, 2). All six species in the *Etheostoma chermocki* complex, which were previously delimited using genomic data [30], are endemic to streams in the Mobile River drainage and have been recognized as imperiled species [34,38–40]. The ranges of these new species flank the Birmingham metropolitan area in Alabama, USA (figure 1*a*,*b*,*c*), and we highlight the conservation implications of the restricted ranges of these species, including their documented local extirpation. Together with the critically endangered vermilion darter *Etheostoma chermocki* Boschung, Mayden and Tomelleri, the vulnerable Warrior darter *E. bellator* Suttkus and Bailey [38,41–44], and the recently described *E. kimberlae* and *E. michellae* [45], for which we also provide differential diagnoses, the new species form a clade of snubnose darters that share common ancestry approximately 5 million years ago [30]. The description of these endangered species of freshwater fishes highlights the value of deploying genomic resources to discover, delimit, describe and protect imperiled species.

## 2. Methods

### (a) Meristic characters

We collected meristic data from 376 specimens obtained from field sampling and museum collections (electronic supplementary material, table S1) and took photographs of living female and male specimens in nuptial condition to assess coloration differences among the lineages delimited as species using genomic analyses [30]. We counted the numbers of scale rows and fin elements from each specimen [46,47] and performed principal component analysis (PCA) of the meristic traits using the ‘prcomp’ function in R v. 3.2.0 (http://www.R-project.org/).

### (b) Genomic species delimitation

Our delimitation of six species in the *E. chermocki* complex is based on phylogenomic and species delimitation analyses of 25 393 double digest restriction-site associated DNA (ddRAD) loci sampled from 67 specimens [30]. The consistent phylogenetic resolution of six lineages in the *E. chermocki* complex and the delimitation of these lineages as species with moderate to high genealogical divergence index (*gdi*) [30] values is supported by estimates of the pairwise fixation index (*F_ST_*) [48] obtained using the R package ‘hierfstat’ v. 0.4.22 [49]. We apply the general lineage species concept [50,51], which defines species as separately evolving metapopulation lineages. Operationally, it is expected that individuals of distinct species will resolve as reciprocally monophyletic groups in a molecular phylogeny and exhibit limited gene flow with other species [52,53]. We highlight diagnostic phenotypic differences among the delimited species of the *E. chermocki* complex.

### (c) Assessment of imperilment

The biological conservation status of the species of the *E. chermocki* complex is based on assessments from the IUCN Red List and fish biologists [34,38–40,54]. In addition, a trend of decline in populations and restricted geographic distributions (e.g. microendemism) are criteria for assessment of extinction risk in freshwater animals that we apply to the species described here [3].

## 3. Results

### (a) Taxonomy

#### (i) Etheostoma kimberlae

Locust Fork darter

YPM ICH 038287 (YFTC 47413), YPM ICH 037410, six females 42.5−52.7 mm SL and eight males 39.5−54.8 mm standard length (SL), Mill Creek at Davidson Road, Blount Co., Alabama, USA (33.9194 N, 86.5270 W), 29 May 2023. YPM ICH 037347, four females 47.6−52.9 mm SL and three males 52.0−58.7 mm SL, Calvert Prong Little Warrior River at Blount County Road 39, Blount County, Alabama, USA, 29 May 2023. UAIC 10867.06, four females 43.1−46.5 mm SL and four males 44.9−46.4 mm SL, Mill Creek at old AL Highway 75, Blount Co., Alabama, USA, 16 June 1993. UAIC 03804.07, five females 34.7−39.6 mm SL and five males 39.0−43.4 mm SL, same locality as UAIC 10867.06, 13 February 1970.

*Referred specimens.* YPM ICH 038287 (YFTC 47413), YPM ICH 037410, six females 42.5−52.7 mm SL and eight males 39.5−54.8 mm standard length (SL), Mill Creek at Davidson Road, Blount Co., Alabama, USA (33.9194 N, 86.5270 W), 29 May 2023.YPM ICH 037347, four females 47.6−52.9 mm SL and three males 52.0−58.7 mm SL, Calvert Prong Little Warrior River at Blount County Road 39, Blount County, Alabama, USA, 29 May 2023. UAIC 10867.06, four females 43.1−46.5 mm SL and four males 44.9−46.4 mm SL, Mill Creek at old AL Highway 75, Blount Co., Alabama, USA, 16 June 1993. UAIC 03804.07, five females 34.7−39.6 mm SL and five males 39.0−43.4 mm SL, same locality as UAIC 10867.06, 13 February 1970.

*Diagnosis and description*. This species was recently erected in [45]. However, the diagnosis of this species in that paper was made with reference to a paraphyletic concept of *E. bellator*, and therefore cannot be used to diagnose the species relative to its closest relatives in the complex. We provide the following as a revised diagnosis. *Etheostom*a *kimberlae* differs from all other species of the *E. chermocki* complex in the presence of fixed allozyme allelic differences of aspartate transaminase, acid phosphatase and adenosine deaminase [42], and pigmentation and coloration patterns of nuptial males (figure 2). *Etheostoma kimberlae* lacks pigment surrounding each lateral line pore, a pattern that forms a narrow horizontal stripe that is less than half a scale-row wide (figure 2). The horizontal stripe in other species of the complex except *E. chermocki* is 1–2 scale-rows wide and extends at least three quarters of the way of the lateral band [33,42] (figures 1*c* and 2). *Etheostoma kimberlae* differs from *E. chermocki* in possessing a ventrolateral vermilion-coloured scale band that does not extend upwards to dark lateral blotches versus extending upwards to dark lateral blotches in *E. chermocki* [43]. *Etheostoma kimberlae* has an average of 47.0 lateral line scales, modally 10 dorsal fin spines and modally 14 pectoral fin rays (table 1). The largest examined male specimen is 58.7 mm SL and the largest female is 47.9 mm SL.

**Table 1.** Comparative meristics and genealogical divergence indices for the new darter species. Average number of lateral line scales, modal number of first dorsal fin spines, modal number of pectoral fin rays and mean value of genealogical divergence index (*gdi*) with 95% highest posterior density (HPD) intervals for *Etheostoma chermocki*, *Etheostoma bellator*, *Etheostoma birminghamense*, *Etheostoma gurleyense*, *Etheostoma michellae* and *Etheostoma kimberlae*.

species	lateral line scales	dorsal fin spines	pectoral fin rays	*gdi* (95% HPD)
*Etheostoma chermocki*	45.82	11	14	0.84 (0.82−0.86)
*Etheostoma bellator*	48.07	10	14	0.49 (0.45−0.52)
*Etheostoma birminghamense*	49.25	10	13	0.49 (0.42−0.58)
*Etheostoma gurleyense*	49.34	10	14	0.65 (0.61−0.68)
*Etheostoma michellae*	47.88	10	13	0.72 (0.68−0.75)
*Etheostoma kimberlae*	46.96	10	14	0.81 (0.79−0.83)

*Geographic distribution*. *Etheostoma kimberlae* is endemic to the Little Warrior River system with populations in Mill Creek and Calvert Prong Little Warrior River in Blount County, Alabama. One specimen was collected in 1969 from Little Cove Creek in the upper Locust Fork system in Etowah County, Alabama, USA (figure 1*a*).

*Conservation note*. Known from only two populations in Mill Creek and Calvert Prong Little Warrior River, the Little Warrior darter (*E*. *kimberlae*) warrants high conservation concern due to its restricted distribution within a river system degraded by agricultural and urban runoff (table 2). Sedimentation from these human activities continues to threaten the species throughout its limited range [34,40]. The species is extirpated from Little Cove Creek (figure 1*a*).

**Table 2.** Land cover percentage in catchment area of each species. The proportion of land cover within each of the catchments (defined at the HUC12 level) where the species are distributed. Land cover information is from the 2023 national land cover dataset [37] (figure 1*b*). Developed includes all categories (open to high intensity) and forest includes mixed, deciduous and evergreen.

species	per cent developed	per cent forest	per cent pasture	per cent other
*Etheostoma kimberlae*	12.1	57.2	25.9	4.8
*Etheostoma gurleyense*	14.4	67.1	13.8	4.7
*Etheostoma michellae*	2.5	94	1.8	1.8
*Etheostoma birminghamense*	51.9	36.6	3.8	7.6
*Etheostoma bellator*	9.3	61.3	23.3	6.1
*Etheostoma chermocki*	29.3	60	7.8	2.8

#### (ii) *Etheostoma gurleyense* new species

Gurley darter

*Zoobank registration*: urn:lsid:zoobank.org:act:86C841F6-C219−48CF−8E54−8ACCDD85CFC7

*Holotype*. YPM ICH 038288, YFTC 47475, adult male, 48.4 mm SL, Gurley Creek at Remlap Drive, Blount Co., Alabama, USA (33.7694 N, 86.6331 W), 30 May 2023.

*Paratopotype.* YPM ICH 037440, 10 females 37.9−55.0 mm SL and nine males 43.7−47.7 mm SL, collected with the holotype.

*Paratypes.* YPM ICH 035286, two females 39.2−46.9 mm SL and one male 45.6 mm SL, collected at the same locality as the holotype, 12 March 2022. UAIC 06364.06, 15 females 33.5−43.0 mm SL and 12 males 37.9−52.9 mm SL, Gurley Creek at AL Highway 75, Blount Co., Alabama, USA, 6 March 1981. UAIC 10447.01, two females 45.6−48.5 mm SL and seven males 41.1−48.2 mm SL, same locality as UAIC 06364.06, 31 July 1992.

*Diagnosis and description*. *Etheostoma gurleyense* differs from all other species of *E. chermocki* complex in the presence of a rare allozyme allele of pyruvate kinase [42], pigmentation and coloration patterns of nuptial males (figure 2). Ventrolateral vermilion-coloured band is 1−2 scale-rows wide and occasionally interrupted (figure 2). *Etheostoma gurleyense* has an average of 49.3 lateral line scales, modally 10 dorsal fin spines, and modally 14 pectoral fin rays (table 1). The largest reported specimens are 60.2 mm SL for males and 58.9 mm SL for females [55].

*Geographic distribution*. *Etheostoma gurleyense* is endemic to a 9.8 km Gurley Creek stretch in Blount County, Alabama, inhabited by shallow, moderate-flow areas with bedrock and gravel or cobble substrate [55].

*Conservation note*. The Gurley darter (*E. gurleyense*) warrants highest conservation concern as the species faces multiple threats within its extremely restricted range in Gurley Creek that lies adjacent to the greater Birmingham metropolitan area (figure 1*b*). Historical museum collection records show a consistent presence of the species from the mid-1960s through mid-2000s, but recent field observations suggest dramatically reduced population densities. The primary threats include water quality degradation from urban and industrial pollution, sedimentation from agricultural runoff, urban development and strip mining for coal [34,56]. Given the species’ highly localized distribution, a single toxic spill could prove catastrophic, potentially leading to extinction. An urgent, systematic survey is needed to assess current population status throughout its very limited geographic range.

#### (iii) Etheostoma michellae

Sipsey Fork darter

*Referred Material.* YPM ICH 038289, YFTC 47543, adult male, 48.1 mm SL, YPM ICH 037338, eight females 29.4−47.9 mm SL and nine males 34.3−43.6 mm SL, Flannagin Creek at NW Road (Lawrence County Road 7), Lawrence Co., Alabama, USA (34.3389 N, 87.3882 W), 1 June 2023; YPM ICH 018574, one female 35.0 mm SL and one male 38.0 mm SL, Sipsey Fork at Winston County Road 60, Winston Co., Alabama, USA, 29 July 2007. YPM ICH 032152, two females 35.0−36.0 mm SL and four males 36.5−38.0 mm SL, Borden Creek at Lawrence County Road 9, Lawrence Co., Alabama, USA, 9 June 2018. UAIC 03851.11, one female 37.5 mm SL and 15 males 29.5−39.3 mm SL, Sipsey Fork, approximately 4 km W of Grayson, approximately 16 km NNE of Double Springs, Winston Co., Alabama, USA, 29 October 1971. UAIC 11065.05, 16 females 27.5−42.3 mm SL and three males 40.1−46.0 mm SL, same locality as YPM ICH 032152, 18 April 1994.

*Diagnosis and description*. This species was recently erected in [45]. However, the diagnosis of this species in that paper was made with reference to a paraphyletic concept of *E. bellator*, and therefore cannot be used to diagnose the species relative to its closest relatives in the complex. We provide the following as a revised diagnosis. *Etheostoma michellae* differs from all other species of *E. chermocki* complex in the presence of a fixed allozyme allelic difference of esterase [42], pigmentation and coloration of nuptial males. Males of *Etheostoma michellae* differ from males of *E. bellator*, *E. birminghamense*, *E. chermocki*, *E. gurleyense* and *E. kimberlae* in having an incomplete ventrolateral vermilion colour band restricted to the width of one scale and from males of *E. kimberlae*, *E. gurleyense*, *E. bellator* and *E. birminghamense* in having a fully turquoise coloured anal fin (figure 2). *Etheostoma michellae* has an average of 47.9 lateral line scales, modally 10 dorsal fin spines and modally 13 pectoral fin rays (table 1). The largest examined male specimen is 52.1 mm SL and the largest female is 47.9 mm SL.

*Geographic distribution*. *Etheostoma michellae* is endemic to the upper Sipsey Fork in the Black Warrior River system. Populations occur in the mainstem of the Sipsey Fork as well as tributaries that include Borden Creek, Caney Creek, Flannagin Creek, North Fork Caney Creek, and Thompson Creek in Lawrence and Winston Counties, Alabama, USA.

*Conservation note*. The Sipsey Fork darter (*E*. *michellae*) warrants high conservation concern due to its limited range in the upper Sipsey Fork watershed. *Etheostoma michellae* has the largest range and lowest exposure to anthropogenic activities relative to other species in the *E. chermocki* complex (table 2), but population surveys in the mid-2000s revealed significant declines in abundance compared to historical collection records, further emphasizing the species vulnerable status [33,57].

#### (iv) *Etheostoma birminghamense* new species

Birmingham darter

*Zoobank registration*: urn:lsid:zoobank.org:act:B7B7EB09−9E00−438F-A7B9−149F7546AE1E

*Holotype*. YPM ICH 038290, YFTC 47504, adult male, 52.8 mm SL, Blue Creek at Johns Road, Jefferson Co., Alabama, USA (33.3864 N, 87.0805 W), 30 May 2023.

*Paratopotype*. YPM ICH 037373, four females 38.8−42.2 mm SL and five males 48.0−52.4 mm SL, collected with the holotype.

*Paratypes*. YPM ICH 033230, three females 36.0−39.5 mm SL, Valley Creek at 25th Ave just south of intersection with 19th Street, Bessemer, Jefferson Co., Alabama, USA, 4 June 2019. YPM ICH 033237, three females 36.5−38.0 mm SL, same locality as holotype and paratopotype, 4 June 2019. YPM ICH 035273, four females 41.5−49.0 mm SL and two males 48.0−51.5 mm SL, same locality as holotype and paratopotype, 21 March 2022. UAIC 03041.15, five females 47.4−51.1 mm SL and six males 44.3−56.1 mm SL, Five Mile Creek at US Highway 11, Jefferson Co., Alabama, USA, 15 August 1968. UAIC 10450.01, 10 females 39.8−45.8 mm SL and 47.2−51.0 mm SL, Five Mile Creek at Hercules Powder Plant, Jefferson Co., Alabama, USA, 6 August 1992.

*Diagnosis and description*. *Etheostoma birminghamense* differs from all other species of *E. chermocki* complex in the presence of a unique allozyme allelic combination of isocitrate dehydrogenase and malate dehydrogenase [42], modally 13 pectoral fin rays, lateral blotches on the anterior half of the body that are taller than wide (table 1; figure 2). *Etheostoma birminghamense* has an average of 49.3 lateral line scales and modally 10 dorsal fin spines (table 1). The largest examined male specimen is 56.1 mm SL and the largest female is 51.1 mm SL.

*Geographic distribution*. *Etheostoma birminghamense* is endemic to the upper portion of the Valley Creek system, a direct tributary of the Black Warrior River. Populations of *E. birminghamense* are limited to a small portion of the mainstem of Valley Creek near Bessemer, Alabama, and Blue Creek, with historical collections through an approximately 7.5 km stretch of Fivemile Creek all in the greater Birmingham metropolitan area in Jefferson County, Alabama, USA.

*Conservation status*. The Birmingham darter (*E. birminghamense*) warrants the highest conservation concern status due to its critically imperilled condition and high extinction risk. The species now persists only in short reaches of Valley Creek and Blue Creek; *E. birminghamense* is extirpated from Fivemile Creek [34] where it was consistently documented from 1966 through 2006. Within the extremely restricted range of the Birmingham darter, 52% of the area is developed (table 2) and the species faces severe threats from multiple sources: water quality degradation from domestic, urban and industrial pollution; sedimentation from agricultural run-off and urban development; and habitat destruction from strip mining for coal [34,56]. An urgent systematic survey is needed to assess the status of remaining populations throughout the restricted range of *E. birminghamense*.

### (b) Species delimitation

Phylogenomic analyses unambiguously resolve six distinct lineages within the *E. chermocki* species complex. One area of uncertainty in the phylogeny is the monophyly of a clade containing *E. bellator* and *E. birminghamense* [33,52] (figure 1*c*); coalescent-based species trees alternatively suggest that *E. birminghamense* is sister to a clade containing *E. michellae* and *E. bellator* [30].

The *gdi* is particularly useful for assessing whether populations are oversplit, as this metric incorporates both gene flow and isolation [58,59], and values <0.2 are indicative of divergence among populations and those greater than 0.5 or 0.7 signal unambiguous metapopulation divergence and thus comparisons among species [59–61]. The six species within the *E. chermocki* complex show *gdi* values ranging from 0.49 to 0.84 (table 1), which are comparable to values of *gdi* estimated for 24 other darter species sampled across 12 sister species pairs [53].

Genetic divergence of the ddRAD loci supports the delimitation of six species in the *E. chermocki* complex as distinct. Weir & Cockerham’s pairwise fixation index (*F_ST_*) is the ratio of the variance between populations to the variance in allele frequencies across populations [48]. Among species of the *E. chermocki* complex, pairwise *F_ST_* ranges between 0.841 and 0.432, with *F_ST_* of 0.475 for the sister species pair *E. bellator* and *E. birminghamense* and 0.606 for the *E. gurleyense* and *E. michellae* sister species pair (electronic supplementary material, table S1; *p* < 0.001 for all values). These high *F_ST_* values are consistent with what is expected among distinct species and indicate a near lack of gene flow among the allopatric lineages that we delimit and describe as species [53,62,63].

Members of the *E. chermocki* species complex exhibit subtle phenotypic differences relative to the deep (>1 million year) divergences among them. While the PCA shows appreciable overlap in meristic morphospace (figure 1*d*), all species show diagnostic differences in coloration (figure 2), and three of the species exhibit diagnostic meristic differences (table 1 and electronic supplementary material, tables S3–S5; figure 1*c*). *Etheostoma kimberlae* and *E. chermocki* have the lowest numbers of lateral-line scales, while *E. gurleyense* and *E. birminghamense* have the highest (table 1; electronic supplementary material, table S3). *Etheostoma chermocki* has modally 11 dorsal fin spines, whereas all other species in the complex have 10 dorsal fin spines (table 1, electronic supplementary material, table S4). Furthermore, both *E*. *michellae* and *E. birminghamense* have 13 pectoral fin rays, but all other species possess 14 pectoral fin rays (table 1; electronic supplementary material, table S5). While the phenotypic differences among these species are subtle, these variations in coloration and meristic counts are consistent with species delimited using phylogenomic analyses (figure 1*c*,*d*; table 1) [30].

## 4. Discussion

The possibility that geographically disjunct populations of *E. bellator sensu lato* represent distinct and undescribed microendemic species has been recognized for several decades [10,33,34,42,64]. However, these species were only recently delimited by leveraging genome-wide DNA sequences of ddRAD loci [30]. Speciation in the *E. chermocki* complex appears to have taken place as populations dispersed to regions of the Sipsey Fork, Little Warrior River and Locust Fork systems after sandstone bedrock caps eroded and exposed underlying carbonate rock units that represent favourable habitat for species in the complex [30]. Our analyses of coloration and meristic characters among lineages in the *E. chermocki* complex reveal differences consistent with the delimitation of six species (figure 1*c*,*d*; table 1).

Previously, all of the new species described here were classified as the Warrior darter *E. bellator*, which is listed as Vulnerable by the International Union for Conservation of Nature (IUCN) and designated as a species of High Conservation Concern by the Alabama State Wildlife Action Plan [34,39]. While this paper was in proofs, the Sipsey Fork and Little Warrior lineages were described as *E. michellae* and *E. kimberlae*, respectively, which rendered *E. bellator* paraphyletic without justification [45]. Several misspellings of the proposed species names in that paper (including in fig. 5 of [45]) also muddle the taxonomic acts and create junior subjective synonyms for these taxa; we recognize *E. michellae* and *E. kimberlae* as valid species based on their appearance first in the text of [45].

Both new species that we describe here and *E. michellae* and *E. kimberlae* are critically imperiled; *E. birminghamense* and *E. gurleyense* are at the highest risk of extinction, while *E. kimberllae* and *E. michellae* warrant high conservation concern due to their limited geographic ranges and ongoing habitat degradation due to human activities [33,34]. Currently, the vermilion darter *E. chermocki* is the only species in the complex with designated critical habitat and protection under the US Endangered Species Act [65].

The urgent need for formal conservation assessment of the four new species is underscored by mapping land cover around Birmingham and Oneonta, Alabama (figure 1*b*). Medium- to high-intensity land development associated with the Birmingham metropolitan area occurs throughout the entire range of both *E. birminghamense* and *E. gurleyense* (figure 1*b*). Of particular concern, *E. birminghamense* has been extirpated from Fivemile Creek [34], with no recent observations despite a consistent presence in museum collections from 1966 to 2006. *Etheostoma kimberlae* and *E. bellator* face significant habitat threats as their ranges exhibit substantial overlap with urban and agricultural development (figure 1*b*; table 2). In contrast, *E*. *michellae*, though still of high conservation concern, inhabits areas of relatively lower human impact characterized primarily by deciduous forest (e.g. Bankhead National Forest; figure 1*b*; table 2).

The Birmingham, Alabama, metropolitan area harbours an exceptional diversity of imperiled species of freshwater fishes. The federally endangered Cahaba shiner (*Paranotropis cahabae*) and the threatened coal darter (*Percina brevicauda*) are distributed in the mainstem of Locust Fork at the mouths of Turkey and Gurley Creeks [66]. Urban aquatic habitats of Birmingham support several endemic and imperiled species in addition to the newly described *E. birminghamense* and *E. gurleyense*. Three of these are federally endangered species protected under the US Endangered Species Act: the watercress darter (*Etheostoma nuchale*), the vermilion darter (*E. chermocki*) and the rush darter (*E. phytophilum*) [43,67,68]. The watercress darter, described in 1965 [67], is restricted to specialized spring-fed habitats characterized by dense aquatic vegetation, particularly watercress (*Nasturtium officinale*) [69,70]. Both the rush darter and vermilion darter require clear, flowing stream habitats with stable substrate conditions for foraging and reproduction [71,72]. That these microendemic species are restricted to stream environments found within Birmingham’s urban landscape highlights the critical importance of protecting these unique aquatic systems [73].

In the era of widespread genome sequencing, we now possess unprecedented opportunities to revisit and refine our understanding of species diversity within imperilled lineages [74,75,76]. The biodiversity crisis is exacerbated by the heightened extinction risk faced by undescribed species [77]. In the southeastern North American freshwater biodiversity hotspot, this phenomenon might be responsible for the loss of numerous taxa, including undescribed cavefishes [78,79], darters and minnows [10,31,80]. The *E. chermocki* complex illustrates how genomic delimitation methods can resolve taxonomic ambiguities in microendemic lineages. The recognition of four new darter species is of paramount conservation significance, given their extremely limited geographic distributions and collective vulnerability to extinction. Moreover, this complex serves as a compelling model system for investigating habitat specialization and mechanisms of allopatric speciation [30]. The preservation of the millions of years of evolutionary history embodied in these snubnose darters—and the broader, species-rich southeastern North American biodiversity hotspot—fundamentally depends on rigorous approaches to integrative species discovery and delimitation.

## Data Availability

All new data are available in the electronic supplementary material associated with this manuscript. See Kim *et al*. [30] for links to the ddRAD data associated with our work on the Etheostoma chermocki complex, including phylogenomic analyses and species delimitation presented in figure 1c. Supplementary material is available online [81].
